# Trimethylornithine Membrane Lipids: Discovered in Planctomycetes and Identified in Diverse Environments

**DOI:** 10.3390/metabo11010049

**Published:** 2021-01-12

**Authors:** Eli K. Moore

**Affiliations:** Department of Environmental Science, School of Earth and the Environment, Rowan University, Glassboro, NJ 08028, USA; mooreek@rowan.edu

**Keywords:** trimethylornithine, intact polar lipids, cell membrane, *Planctomycetes*, northern wetlands

## Abstract

Intact polar membrane lipids (IPLs) are the building blocks of all cell membranes. There is a wide range of phosphorus-free IPL structures, including amino acid containing IPLs, that can be taxonomically specific. Trimethylornithine membrane lipids (TMOs) were discovered in northern wetland *Planctomycete* species that were isolated and described in the last decade. The trimethylated terminal nitrogen moiety of the ornithine amino acid in the TMO structure gives the lipid a charged polar head group, similar to certain phospholipids. Since their discovery, TMOs have been identified in various other recently described northern latitude *Planctomycete* species, and in diverse environments including tundra soil, a boreal eutrophic lake, meso-oligotrophic lakes, and hot springs. The majority of environments or enrichment cultures in which TMOs have been observed include predominately heterotrophic microbial communities involved in the degradation of recalcitrant material and/or low oxygen methanogenic conditions at primarily northern latitudes. Other ecosystems occupied with microbial communities that possess similar metabolic pathways, such as tropical peatlands or coastal salt marshes, may include TMO producing *Planctomycetes* as well, further allowing these lipids to potentially be used to understand microbial community responses to environmental change in a wide range of systems. The occurrence of TMOs in hot springs indicates that these unique lipids could have broad environmental distribution with different specialized functions. Opportunities also exist to investigate the application of TMOs in microbiome studies, including forensic necrobiomes. Further environmental and microbiome lipidomics research involving TMOs will help reveal the evolution, functions, and applications of these unique membrane lipids.

## 1. Introduction—Amino Acid Containing Membrane Lipids

Cell membranes are the interface between biological processes and the environment [1]. Intact polar lipids (IPLs) are the constituent components of the cell membrane lipid bilayer, consisting of a polar head group and an apolar core. The diverse molecular structures of IPLs can provide functional and taxonomic information on the microbial community of a given environment [2,3]. Following cell lysis, the head groups of IPLs are rapidly cleaved from the core lipids, on the order of days, thus making IPLs representative of living biomass [4,5]. The structures of IPL head groups are very diverse, including common phosphoglycerol IPLs [6] (e.g., phosphatidylcholine, PC; phosphatidylethanolamine, PE; etc.), and various bacterial IPLs that have polar head groups containing amino acids [7]. The amino acid IPLs phosphatidylserine and homoserine-containing betaine lipids have a glycerol backbone, while other amino acid containing lipids are glycerol free.

**Figure 1:**
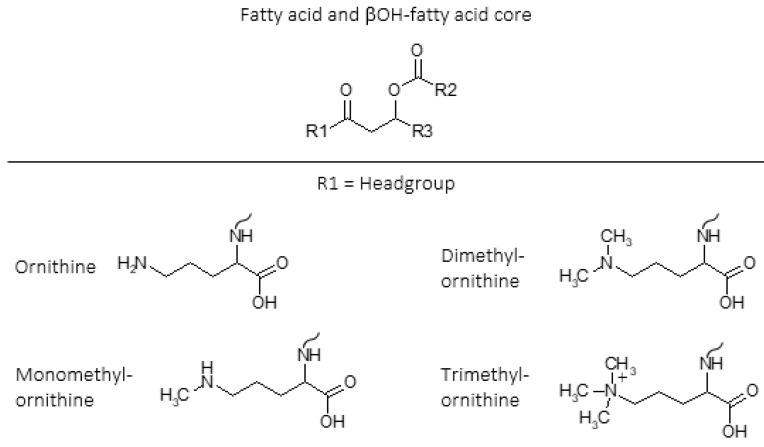
Intact polar lipid (IPL) structures of ornithine (OL), monomethylornithine (MMO), dimethylornithine (DMO), and trimethylornithine (TMO) lipids including fatty acid and βOH-fatty acid core lipids. R1 = headgroup; R2, R3 = alkane/alkene chains. Ornithine (OL; [13,14,15]); monomethylornithine (MMO; [16]); dimethylornithine (DMO; [16]); trimethylornithine (TMO; [16]). Figure adapted from [17].

Ornithine lipids (OLs) are a common group of glycerol free amino acid containing lipids in bacteria (Figure 1). Approximately 50% of bacteria whose genomes have been sequenced are predicted to have the capability to synthesize OLs, but the genes necessary to form OLs are absent in archaea and eukaryotes [7,8,9,10]. The OL head group consists of an amino acid that is linked by an amide bond to a β-hydroxy fatty acid, and the β-hydroxy fatty acid is “piggy back” esterified to a fatty acid (Figure 1). Additionally, lysine containing membrane lipids synthesized by *Agrobacterium tumefaciens* [11] and *Pedobacter saltans* [12] have the same ester linked fatty acid moiety and amide linked β-OH-fatty acid fatty acid structure that exists in OLs.

Ornithine lipids do not contain phosphorus (Figure 1), and can be produced by certain bacteria as an alternative to phosphoglycerol lipids in response to phosphorus limitation [18,19]. Ornithine lipid head groups and the fatty acid groups can also be hydroxylated in response to different environmental stress conditions [20,21]. It has been proposed that the hydroxylation of OLs increases hydrogen bonding between lipid molecules, thus providing enhanced membrane fluidity and stability [22]. Similarly, it was discovered that lysine lipids (LLs) can be hydroxylated on both the head group and fatty acid chains in response to temperature and pH stress in the soil bacteria *Pedobacter saltans* [12]. While various other microbial membrane lipids can be produced or modified in response to changing environmental conditions, OLs and other glycerol-free amino acid lipids are commonly observed to be involved in stress response [9].

In 2013, three novel classes of amino acid containing membrane lipids were discovered in multiple *Planctomycetes* that were isolated from ombrotrophic (receives water only from rain) northern wetlands in European north Russia: mono-, di-, and trimethylated ornithine lipids ([16]; Figure 1). In particular, the unique physical properties of trimethylated ornithine lipids (TMOs) appear to be adapted to the acidic, low nutrient, and anoxic conditions of ombrotrophic northern wetlands. Shortly thereafter, TMOs were identified in various other diverse environments that are often characterized by unique physical or chemical conditions. This review will summarize the discovery of TMOs, their physical and chemical properties, potential functions, and distribution in diverse environments.

## 2. Methylated Ornithine Lipids Discovered in Northern Wetland *Planctomycetes*

In recent decades, there has been great interest in the changing conditions of Northern peatlands due to warming temperatures resulting in enhanced decomposition of stored organic carbon and greenhouse gas emissions [23,24,25,26]. Northern peatlands comprise a significant global carbon store, containing roughly one third of global soil organic carbon while only taking up just 3% of the Earth’s land area [27,28]. These low-temperature systems are ombrotrophic, meaning that they are rain fed (only receive water from rain), nutrient poor, and acidic. Microbial communities are the terminal degraders of organic carbon and producers of greenhouse gases from soils and peatlands [29]. Therefore, understanding the wide range of microbial species and communities in Northern peatlands that are involved in carbon degradation and greenhouse gas production is crucial for understanding the impacts of climate change on global terrestrial carbon storage [30,31].

Given that amino acid containing IPLs are often synthesized or modified by bacteria in response to environmental stress conditions, they can be potential biomarkers in rapidly changing ecosystems. The microbiology research group led by Svetlana Dedysh has significantly advanced our understanding of Northern peatland microbial communities, including the discovery of numerous microbial species isolated from these environments [32,33,34,35,36]. The bacterial phylum *Planctomycetes* were found to be highly abundant in Northern peatlands, and appear to play an important role in the decomposition of the *Sphagnum* moss-derived vegetation litter that dominates these environments [32,36,37,38].

**Figure 2:**
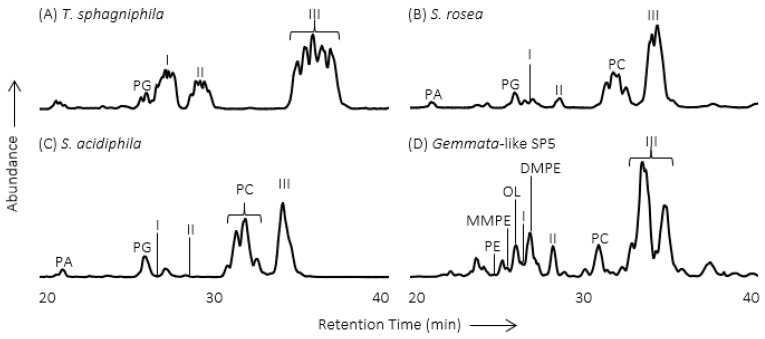
High-performance liquid chromatography-mass spectrometry (HPLC-MS) chromatograms (*m*/*z* 400 to 2000) of Bligh and Dyer lipid extracts from Planctomycete cultures. (**A**) T. sphagniphila; (**B**) S. rosea; (**C**) S. acidiphila; (**D**) Gemmata-like strain SP5. PG = phosphatidylglycerol; PA = phosphatidic acid; PC = phosphatidylcholine; PE = phosphatidylethanolamine; MMPE = monomethyl-phosphatidylethanolamine; DMPE = dimethyl-phosphatidylethanolamine; OL = ornithine lipid; I = monomethylornithine; II = dimethylornithine; III = trimethylornithine. Figure adapted from [16].

In order to understand how Northern peatland *Planctomycetes* are suited to live in these unique conditions, and how they may respond to environmental change, the membrane lipids were analyzed from three *Planctomycete* species (*Singulisphaera acidiphila*, *Singulisphaera rosea*, *Telmatocola sphagniphila*) and one *Gemmata*-like *Planctomycete* strain that were all isolated from peatlands of European north Russia [16]. Initial analysis by liquid chromatography-mass spectrometry (LC-MS) resulted in the observation of three unknown IPL classes in each species and strain (Figure 2). The MS fragmentation pattern of the unknown IPLs is similar to OLs, including the sequential loss of a hydroxy fatty acid and a fatty acid, but with a different MS^3^ product ion *m/z* value.

The most abundant IPL in *T. sphagniphila* (peak III from Figure 2A) was purified using LC-MS with fraction collection for further analysis using nuclear magnetic resonance (NMR) and high-resolution mass spectrometry (HRMS). Analysis using both techniques confirmed that the most abundant unknown IPL in *T. sphagniphila* was an OL that was trimethylated on the terminal nitrogen of the IPL head group (Figure 1; MS fragmentation and NMR results are described in detail by [16]). With this knowledge in hand, HRMS analysis of the lipid extracts from the three *Planctomycete* species and *Gemmata*-like strain confirmed the other unknown IPL classes I and II (Figure 2) to be ornithine lipids that were monothylated and dimethylated on the terminal nitrogen of the IPL head group, respectively (Figure 1). Additionally, similar fatty acid distributions between the monomethylornithine, dimethylornithine, and trimethylornithine lipids (MMOs, DMOs, and TMOs) within each species suggest that the three lipid classes are biosynthetically linked. The most abundant core lipids observed in MMOs, DMOs, and TMOs among the analyzed *Planctomcyetes* were C_18:1_ and C_16:1_ for *T. sphagniphila*, C_18:1_ and C_18:0_ for *S. rosea*, C_18:1_ and C_18:0_ for *S. acidiphila*, and C_20:1_ and C_16:0_ for the *Gemmata*-like strain SP5 [16].

The sequential methylation of the terminal nitrogen among MMOs, DMOs, and TMOs is analogous to the sequential methylation of phosphatidylethanolamine (PE) by the enzyme phosphatidylethanolamine N-methyltransferase, producing monomethylphosphatidylethanol-amine (MMPE), dimethylphosphatidylethanolamine (DMPE), and ultimately the very common IPL phosphatidylcholine (PC) [39,40,41]. The OL N-methyltransferase OlsG (Sinac_1600) gene is responsible for the three-fold methylation of the terminal δ-nitrogen of OL as shown in the TMO producing *S. acidiphila* [42]. The trimethylated terminal nitrogen of PCs gives them a positively charged quadruple bonded nitrogen, and a cylindrical shape compared to the conical shape of PEs, the un-methylated counterparts of PCs. Due to their polarity and relatively cylindrical shape, PCs spontaneously form lipid bilayers, while the cone shape of PEs causes them to assemble into inverted hexagonal phases. Similarly, the additional three methyl groups on the terminal nitrogen in the head group of TMOs may also give these IPLs a more cylindrical shape and greater polarity than un-methylated ornithine lipids and thus result in greater bilayer stability [16]. It has been proposed that OLs are important for outer membrane stability in Gram-negative bacteria due to their zwitterionic nature [43]. However, the enhanced polarity of the positively charged quadruple bonded nitrogen in TMOs could provide greater membrane stability in the cold acidic conditions of ombrotrophic Northern peatlands without using scarce phosphate in these nutrient limited environments.

## 3. High Abundance of TMOs at the Oxic/Anoxic Interface of Northern Wetlands

Following the discovery of monomethylornithine, dimethylornithine, and trimethylornithine lipids, a peat core was analyzed from the Obukhovskoye Bog (58°14′ N, 38°12′ E), approximately 200 miles northeast of Moscow, Russia, in order to search for these novel methylated ornithine lipids and observe the importance of *Planctomycetes* in Northern peatland microbial communities. The peat core had been collected and microbial community of the core had been described in previous studies [34,44]. The peat core was divided into five sections (5 to 10 cm, 10 to 20 cm, 20 to 30 cm, 30 to 40 cm, and 40 to 50 cm) and IPLs were extracted from Obukhoskoye peat using a modified Bligh and Dyer extraction method [45,46]. Intact polar lipid extracts of a peat core collected from the Saxnäs Mosse bog (56°51′20.78 N, 13°27′39.62 E) near the village of Lidhult, southwestern Sweden that were previously collected and analyzed for IPLs [47,48], were analyzed again in order to search for methylated ornithine lipids [49]. The Saxnäs Mosse peat core was extracted for IPLs in 2 cm sections from 0 cm to 54 cm. Additionally, genetic analysis was performed on selected samples from both peat cores in order to confirm the presence *Planctomycetes* and compare their sequence abundance with other microbial taxa.

**Figure 3:**
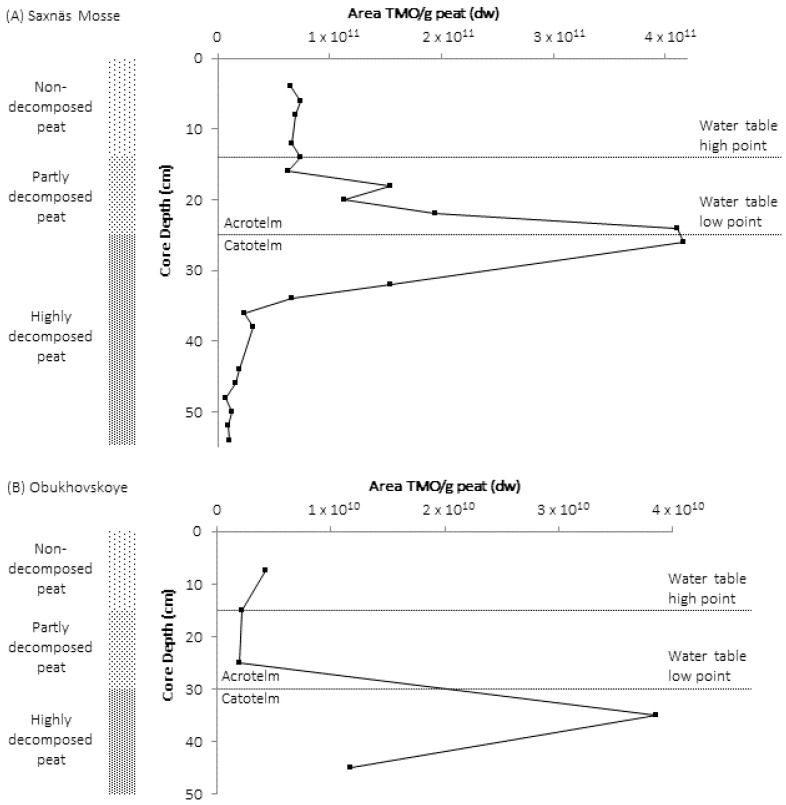
Relative abundances of trimethylornithine IPL (TMO) from HPLC-MS analysis down core in Saxnäs Mosse peat (**A**) and Obukhovskoye peat (**B**). dw, dry weight. Figure adapted from [49].

Trimethylornithine lipids were present in high abundance throughout the peat core depth profiles of both the Obukhovskoye and Saxnäs Mosse bogs, and the TMO concentration peaked at the oxic/anoxic interface in both cores ([49]; Figure 3). The peak in TMO concentration coincided with the maximum abundance of *Planctomycete*-specific 16S rRNA gene sequences. *Planctomycete* gene sequences detected at the oxic/anoxic interface were affiliated with the *Isosphaera* group, while sequences present in the anoxic peat layers were related to an uncultured *Planctomycete* group. Pyrosequencing identified *Planctomycetes* as the major bacterial group at the oxic/anoxic interface in Obukhovskoye peat (54% of total 16S rRNA gene sequence reads), followed by *Acidobacteria* (19% reads), while in the Saxnäs Mosse peat, *Acidobacteria* were dominant (46%), and *Planctomycetes* contributed to 6% of the total reads. The detection of abundant TMO lipids in *Planctomycetes* isolated from peat bogs [16] and the absence of TMO production by all known cultured species of *Acidobacteria* suggest that *Planctomycetes* are the producers of TMOs in the peat bogs.

The distribution of TMO core lipids number of carbons and double bonds changed throughout the Obuhovskoye and Saxnäs Mosse peat cores, which likely reflects the change in *Planctomycete* community reflected by gene sequences with depth. In the Saxnäs Mosse Bog core, the TMO lipid with C_18:1_ and βOH-C_19:0_ core lipids accounted for >60% of total TMOs at the oxic/anoxic interface depth; the TMO with C_19:2_ and βOH-C_18:0_ core lipids and the TMO with C_18:2_ and βOH-C_18:0_ core lipids were most abundant just above and just below the oxic/anoxic interface; and the TMO with C_19:1_ and βOH-C_16:0_ core lipids and the TMO with C_18:1_ and βOH-C_18:0_ core lipids were most abundant at the peat surface [49]. In particular, the *Isosphaera* cluster was the most abundant *Planctomycete* group at the oxic/anoxic interface. Due to this observation, the *Isosphaera*-like strain PX4, which was isolated from the Obukhovskoye bog just above the oxic/anoxic interface, was cultured at oxic (7 mg/L) and micro-oxic (1.5 mg/L) conditions. The relative production of TMOs increased in PX4 grown in micro-oxic conditions, including a dramatic increase in concentration of TMO with C_19:1_ and βOH-C_18:0_ core lipids. The increase in TMOs and shift in TMO core lipids in micro-oxic conditions, the higher accumulation of TMOs at the oxic/anoxic interface in Northern wetlands, and the change in the *Planctomycete* community with depth suggest that these IPLs could be synthesized as a response to low oxygen levels and changing redox conditions at the oxic/anoxic interface. Anoxic peat and soils are important environments for the global production of methane [50,51], and TMOs may be an important factor allowing *Planctomycetes* to be involved in organic matter degradation and subsequent methane production by the microbial community of micro-oxic and anoxic peatlands.

## 4. Identification of TMOs in Diverse Environments

**Figure 4:**
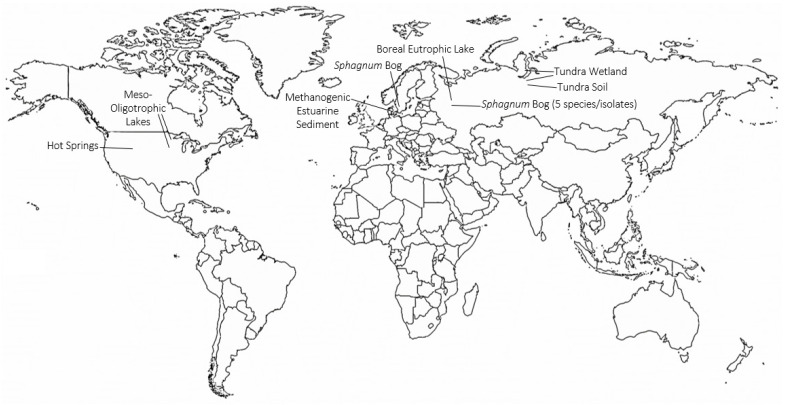
Global map showing locations and ecosystems where trimethylornithine lipids have been identified. Global map made with mapchart.net.

Continued work by the Svetlana Dedysh laboratory to characterize the microbial communities of northern latitude environments has led to the identification of TMOs in various recently described *Planctomycete* species (Figure 4). Trimethylornithines are the major membrane lipid constituents of the hydrolytic northern tundra wetland *Planctomycete Paludisphaera borealis* [52], the psychrotolerant *Isosphaeraceae Planctomycete* from lichen-dominated tundra soils *Tundrisphaera lichenicola* [53], the *Sphagnum* peat bog *Planctomycete Fimbriiglobus ruber* of the proposed family *Gemmataceae* [54], the freshwater *Planctomycete Limnoglobus roseus* isolated from a boreal eutrophic lake [55], and the psychrotolerant cellulolytic *Gemmataceae Planctomycete* from a littoral tundra wetland *Frigoriglobus tundricola* [56]. The identification of TMOs in *Planctomycetes* from a northern wetland, tundra soil, peat bog, boreal eutrophic lake, and tundra wetland show that TMOs are more widespread in northern latitude environments than initially thought.

Trimethylornithine lipids have subsequently been identified in a wide range of aquatic environments since their discovery in Northern wetland *Planctomycete* isolates (Figure 4). An extensive study of trophic state impact on IPL distribution in North American lake surface waters from Minnesota and Iowa found the presence of TMOs, OLs, and betaine lipids in meso-oligotrophic lakes [57]. The higher relative abundance of ornithines and TMOs in the observed meso-oligotrophic lakes was proposed to relate to a higher contribution of heterotrophic bacteria relative to phytoplankton in the lakes, similar to the presence of TMOs in abundant heterotrophic *Planctomycetes* of Northern peatlands. Trimethylornithines were identified in the oxic, micro-oxic, and anoxic layers of Northern wetland peat [49], therefore, it is consistent to observe TMOs in oxic lake waters. Conversely, TMOs were later identified in enrichment cultures of anoxic methanogenic sediment from Arhus Bay, Denmark [58], akin to the presence of TMOs in anoxic methanogenic peat. Among the enrichment cultures that contained TMOs, the cultures that received sulfate amendments had higher TMO relative abundances than the TMO-containing enrichment culture that did not receive additional sulfate.

Trimethylornithine lipids were also recently observed in microbial communities of multiple Yellowstone National Park hot springs ([59,60]; Figure 4). The fatty acids of some of the hot spring TMOs were hydroxylated [59]. Ornithine lipids were also observed in the hot springs, and various OLs were found to contain hydroxylated fatty acids as well. It has previously been shown that OL and LL fatty acids can be hydroxylated in response to temperature and pH stress [12,20,21]. The hydroxylated fatty acids of Yellowstone TMOs and OLs may also be a stress response to elevated hot spring temperatures in order to create more hydrogen bonding and membrane stability. The high relative abundance of TMOs in the Yellowstone hot spring microbial undermat suggests that these lipids could originate from abundant anoxygenic phototrophic organisms in these layers; however, the authors suggest a chemoheterotrophic origin [60], which aligns much closer with the known metabolic niches of TMO producing microbes. Given that TMOs have only been identified in *Planctomycete* species, further work characterizing new microbial species in environments where TMOs are observed will help confirm if these lipids are lipid biomarkers for *Planctomycetes*, or if they are a more taxonomically diverse membrane lipid.

## 5. TMOs: Specialized Lipids with Potential Broad Distribution

The distribution of TMOs among different types of ecosystems suggests that this class of lipids may have specialized and flexible functions depending on the surrounding environmental conditions or microbial community. The similar structure of the trimethylated terminal nitrogen of TMOs compared to the choline moiety of PCs, without the presence of phosphorus, suggests that TMOs may have a similar membrane structural role to PCs under phosphorus limiting conditions [16]. The increase of relative TMO production in *Planctomycete* cultures grown under low oxygen conditions [49] and the presence of hydroxylated TMOs in hot spring environments [59] further link TMOs to stress response, similar to the production and modification of other amino acid containing lipids under the nutrient, temperature, or pH stress conditions described above. Additionally, hot spring TMOs are present in high relative abundance at and below the oxic–anoxic interface of the microbial mat, and not present in the oxic upper layer [60]. The increased relative production of TMO lipids in low oxygen cultures, the presence of abundant TMOs in anoxic methanogenic peat, anoxic hot spring microbial mats, and anoxic methanogenic estuarine sediment enrichment cultures further indicate that these lipids may be present in other anoxic and/or methanogenic environments.

In addition to links between TMOs and stress responses, TMOs have been observed in both oxic and anoxic heterotrophic microbial communities in northern peatlands, northern lakes, tundra soils, tundra wetlands, northern eutrophic lakes, and mid-latitude meso-oligotrophic lakes (Figure 4). Future microbial lipidomics studies on aquatic and soil ecosystems at northern and mid-latitudes should include TMOs in their IPL targets to further characterize the microbial community and potential contribution of *Planctomycetes* in their study systems. Such studies in aquatic and soil ecosystems at lower latitudes will be intriguing for potentially identifying TMOs as well, thus expanding the known geographic distribution of these lipids. Similar to high latitude peatlands, tropical peatlands are important global carbon stores, with variable carbon fluxes in relation to land use and climate change [61,62]. *Planctomycetes* have been identified in metabolically diverse tropical peat microbial communities [63], suggesting that TMOs could be important microbial membrane constituents in these environments. Coastal salt marshes also contain diverse microbial communities, including *Planctomycetes* [64], that are involved in the decomposition of recalcitrant organic matter [65,66], that commonly include anoxic and methanogenic sediment zones at depth [67]. The distribution of TMOs and potential modifications of TMOs could inform the role of *Planctomycetes* and adaptations to lower latitude environments, such as tropical peatlands and coastal salt marshes.

A new area of application for the analysis of TMOs and other IPLs is forensics microbiome research [68,69]. Understanding heterotrophic microbial communities is crucial in forensics studies that investigate changing microbiomes of decaying vertebrate remains. Bacterial community succession analysis of the necrobiome associated with decaying vertebrate remains has revealed a negative relationship for overall taxon richness with increasing decomposition [70], and heterotrophic bacteria have also been observed to increase during decomposition of vertebrate remains [71]. Microbial community succession in forest soil below decomposing human cadavers has shown that *Planctomycete* sequence abundance decreased during the bloat-active decomposition stage to the advanced decay II stage, but then returned to original relative abundance during the advanced decay III stage [72]. This suggests that the presence of soil *Planctomycetes* under decayed vertebrate remains could indicate that the advanced decomposition of labile organic matter has taken place allowing Planctomycetes cell numbers to recover. This in agreement with the role of soil *Planctomycetes* to be involved in the degradation of recalcitrant material after labile material has been consumed [37]. In such a scenario, TMOs and other microbial IPLs could be used to track microbiome changes during carrion decay. Further microbiome and environmental lipidomics research involving TMOs in diverse ecosystems will help reveal the evolution, functions, and applications of these unique membrane lipids.
